# Nanopillar Diffraction Gratings by Two-Photon Lithography

**DOI:** 10.3390/nano9101495

**Published:** 2019-10-19

**Authors:** Julia Purtov, Peter Rogin, Andreas Verch, Villads Egede Johansen, René Hensel

**Affiliations:** 1 INM—Leibniz Institute for New Materials, Campus D2 2, 66123 Saarbrücken, Germany; 2 Department of Materials Science and Engineering, Saarland University, 66123 Saarbrücken, Germany; 3 Department of Chemistry, University of Cambridge, Lensfield Road, Cambridge CB2 1EW, UK

**Keywords:** nanostructures, optical pillar gratings, photonic crystals, two-photon lithography, direct laser writing

## Abstract

Two-dimensional photonic structures such as nanostructured pillar gratings are useful for various applications including wave coupling, diffractive optics, and security features. Two-photon lithography facilitates the generation of such nanostructured surfaces with high precision and reproducibility. In this work, we report on nanopillar diffraction gratings fabricated by two-photon lithography with various laser powers close to the polymerization threshold of the photoresist. As a result, defect-free arrays of pillars with diameters down to 184 nm were fabricated. The structure sizes were analyzed by scanning electron microscopy and compared to theoretical predictions obtained from Monte Carlo simulations. The optical reflectivities of the nanopillar gratings were analyzed by optical microscopy and verified by rigorous coupled-wave simulations.

## 1. Introduction

Two-dimensional photonic structures such as periodical pillar gratings are applicable for light coupling devices [1], solar devices [2], sensors [3], encoders [4], holographic structures [5], or security features [6]. The optical characteristics of such gratings are very sensitive to the shape, diameter, and height of the nanostructures, as well as the pitch and periodicity of the array [7,8,9,10,11]. Therefore, these features require strict tolerances to ensure uniformity with virtually no defects within the grating. Simultaneously, there is a desire to enable flexible manufacturing of optical elements, as each application has its own requirements regarding design and feature size. In this context, two-photon lithography (TPL) is a promising candidate for the fabrication of nanostructured gratings with tunable optical properties.

In recent years, TPL has been established as a suitable technique for the fabrication of complex three-dimensional structures with submicron resolution [12,13,14,15]. Its versatility has been demonstrated by various applications ranging from microfluidic devices [16], micropatterned adhesives [17,18], biological and mechano-sensitive scaffolds [19,20,21,22], and optical devices, such as lenses [23] and photonic crystals [24,25]. In a typical TPL process, a focused, femtosecond-pulsed, near-infrared laser (λ = 780 nm) exposes a photoresist, that is composed of reactive oligomers and a photoinitiator. However, the photoreaction is only initiated when two photons excite the initiator concertedly. As a result, the initiator molecule decomposes into radicals, which induce a cross-linking reaction of the oligomers. Such a two-photon event is very rare, so that the probability of a two-photon excitation, and thus the start of polymerization, is only given in the focal region where the photon density is highest [15]. The polymerization reaction is in most cases terminated by oxygen quenching of radicals [26]. The size of the voxel, i.e., the volume element at which the polymerization occurs, is a function of the beam [15,27,28], the exposure parameters [29,30], and the chemical and physical properties of the photoresist [26,29,31,32]. In order to estimate the lateral voxel size S, Sun et al. [33] empirically derived an equation based on the diffraction limit $l_{diff}$, a material constant α, the applied laser power $E_{re}$, and the threshold laser power $E_{th}$ required for the cross-linking reaction:

(1) $$S=l_{diff}\alpha \sqrt{\frac{\ln\left(E_{re}/E_{th}\right)}{4k\ln2}}$$

where k equals 1 or 2 for a single or two-photon excitation, respectively. According to Equation (1), S decreases with $E_{re}$ and reaches 0 for $E_{re}=E_{th}$. From this it can be concluded that the smallest lateral feature sizes are achieved for laser powers close to the polymerization threshold of the photoresist. Small feature sizes, however, are highly susceptible to deformations and collapse due to their low mechanical resistance [34,35,36]. In particular, nanostructures with high aspect ratios and low mechanical stiffness are prone to such defects [37,38].

In the present study, we report on the fabrication of nanopillar diffraction gratings. These are fabricated via TPL and laser energies close to the polymerization threshold of the photoresist (10–23 mW) combined with a recently reported, improved development routine [36]. The obtained structures are analyzed by scanning electron microscopy. To enable a prediction of the size of the nanopillars for further investigations, numerical simulations based on a Monte Carlo algorithm were performed. The optical properties of the gratings with pillar diameters between 120 and 430 nm and heights ranging from 330 to 1315 nm were corroborated by rigorous coupled-wave analysis (RCWA) simulations.

## 2. Experimental

### 2.1. Two-Photon Lithography (TPL)

Diffraction gratings were fabricated from a negative tone photoresist IP-Dip (Nanoscribe, Eggenstein-Leopoldshafen, Germany) on fused silica substrates using the Professional GT two-photon lithography system (Nanoscribe, Eggenstein-Leopoldshafen, Germany). The system consisted of a 63× objective (NA = 1.4, Carl Zeiss, Oberkochen, Germany) and a femtosecond pulsed IR-laser (λ = 780 nm, 80 MHz laser repetition rate, and 100–200 fs pulse length, TOPTICA Photonics AG, Graefelfing, Germany). The system was operated in ‘dip-in’ mode, where the objective is immersed into the photoresist. The nanopillars were arranged with a center-to-center distance of 1 µm in a square lattice of 50 × 50 µm. Each pillar consisted of four vertically stacked voxels, whose focal points were separated by 300 nm, whereby the lowest voxel was placed 200 nm below the substrate-resist interface to ensure appropriate attachment to the fused silica substrate. The exposure and settling times were set to 0.1 and 2 ms, respectively. All diffraction gratings were fabricated on the same substrate with altered laser powers ranging from 10 to 23 mW for different gratings. To improve the mechanical stability of the nanopillars, the development was performed according to Purtov et al. [36]. Structures were developed for 20 min in PGMEA (Sigma-Aldrich, Steinheim, Germany), after which 70% of the solution was carefully replaced with isopropanol (Sigma-Aldrich, Steinheim, Germany) without exposing the structures to air. Such a solvent exchange was repeated three times, separated by a residence time of 10 min. Subsequently, a UV-post-crosslinking was applied (t = 300 s, $\lambda_{UV}$ = 365 nm, 350 mW, OmniCure S1500A, igb-tech, Friedelsheim, Germany) before structures were removed from the liquid and air-dried.

### 2.2. Scanning Electron Microscopy (SEM)

Samples were fixed on a metallic sample holder and investigated at tilt angles of 0° and 40° using a Quanta 250 FEG (FEI, Eindhoven, The Netherlands) equipped with an Everhart-Thornley-Detector (ETD) in high-vacuum mode. Copper tape was placed close to the nanostructures to avoid charging, as no conductive coating was applied to preserve the optical properties of the arrays. The spot size and the acceleration voltage were set to 2.0 and 2 kV, respectively. The measured pillar heights in micrographs were corrected for the sample tilt.

### 2.3. Optical Microscopy

The optical reflection characteristics of the pillar arrays were investigated using an optical microscope (Eclipse LV100ND, Nikon, Tokyo, Japan) equipped with a 20× color-corrected objective (NA = 0.45). The microscope was operated in bright field mode with a fully opened illumination aperture upon a white balance using a white sheet of paper.

## 3. Numerical Simulations

### 3.1. Voxel Sizes

In order to gain a better understanding of the size- and shape-changing effects during the TPL-process, the obtained nanostructures were analyzed and compared to theoretical pillar sizes derived from simulations considering the different laser powers applied. Since the Gaussian beam formalism based on the paraxial approximation is not appropriate to describe the tightly focused beam used in the experiment, the electric field distribution around the focal spot had to be determined by numerical integration. This integration was performed using the Huygens’ principle, i.e., by assuming the field distribution to be the result of superimposing fields originating from an ensemble of emitting elementary sources. This approach was implemented in a self-written software executing a Monte Carlo algorithm.

The coordinate system of the simulation was defined with the light incident from the +z-direction. The origin of the coordinate system was set to the focal spot of the focussed beam. The elementary emitters were assumed to be dipole oscillators distributed in a planar arrangement parallel to the x-y focal plane with a normal distance $z_{0}$. The plane of the dipole oscillators can be regarded as the exit pupil of the focusing objective with $z_{0}$ being the working distance. The phase of the dipoles φ(r) as a function of distance r from the z-axis was adjusted to result in a constant phase at the origin of the coordinate system (i.e., the center of the focal spot), which gives focusing:

(2) $$\varphi =-\frac{2\pi }{\lambda }\cdot \sqrt{z_{0}^{2}+r^{2}}$$

The amplitude of the dipole strength per unit area $\vec{P}$ followed a Gaussian radial profile, while the polarization was assumed to be circular in order to result in a rotationally symmetric field distribution around the focal spot. By arbitrarily setting all constant factors to 1, the full description of the radial distribution of the dipole strength is given by:

(3) $$\vec{P}\left(r\right)=e^{-{\left(r/w\right)}^{2}}\cdot \left(\begin{array}{c} 1\\ i\\ 0 \end{array}\right)\cdot e^{i\varphi \left(r\right)}$$

The width w of the Gaussian function was calculated from the half divergence angle α, (experimentally determined to be 31.4 degrees by analyzing the beam profile as a function of the z-coordinate) and the working distance $z_{0}$, as

(4) $$w=z_{0}\cdot tan\left(\alpha \right)$$

The distribution described above was then cut-off at a finite maximum radius $r_{max}$ representing the finite opening of the focusing lens, which is defined by

(5) $$arctan\left(r_{max}/z_{0}\right)=arcsin\left(\frac{NA}{n}\right)$$

where NA is the numerical aperture of the lens and n the refractive index of the medium.

**Figure 1:**
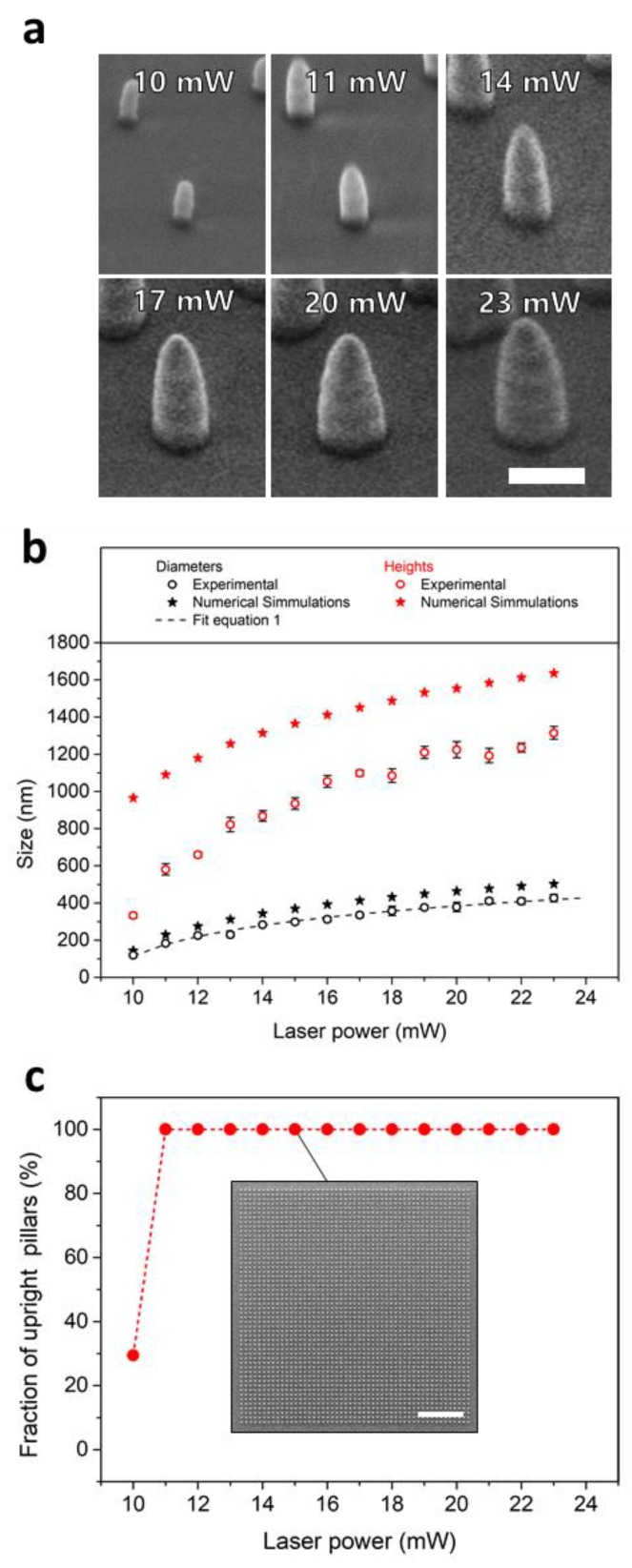
Sizes of nanopillars as a function of the applied laser power. (**a**) Scanning electron micrographs of nanopillars fabricated with different laser powers. The scale bar is 500 nm. (**b**) Diameters (black symbols) and heights (red symbols) of the nanopillars obtained from two-photon lithography (open circles) compared to numerical simulations (filled stars). The dashed line shows the fit of the pillar diameters using Equation (1) to estimate the threshold laser power of the photo resist. (**c**) Defect rates of optical gratings expressed as fractions of upright pillars in dependence on the applied laser power. The values were obtained from Scanning Electron Microscopy (SEM)-images as shown for 15 mW in the insert. The scale bar is 10 µm.

The field distribution near the focal spot was calculated as the superposition of elementary waves emerging from the emitter distribution (Equation (3)). To do this, the field in the volume surrounding the focal spot was mapped to a two-dimensional array of pixels addressed by axial and radial coordinates in the x-z-plane, taking advantage of the deliberately introduced rotational symmetry of the source. The resolution of this field map was chosen to be λ/50, where λ was set to 780/1.52 = 513 nm (the wavelength of the laser divided by the refractive index of the photoresist). The Monte Carlo algorithm repeatedly picked a randomly selected pair of a pixel ${\vec{x}}_{f}$ in this map and a point ${\vec{x}}_{s}$ in the source distribution in order to calculate the contribution of the source emitter to the selected pixel. The latter was taken to be the field of an elementary dipole equivalent to the emitter strength $\vec{P}=\vec{P}\left({\vec{x}}_{s}\right)$. Omitting constant factors, the elementary dipole field is given by:

(6) $$\vec{E}\left({\vec{x}}_{f}\right)=\left\{\frac{\left(\vec{n}\times \vec{P}\right)\times \vec{n}}{R}+\left[3\cdot \vec{n}\cdot \left(\vec{n}\cdot \vec{P}\right)-\vec{P}\right]\cdot \left(R^{-3}+i\cdot R^{-2}\right)\right\}\cdot e^{iR}$$

where $R=\frac{2\pi }{\lambda }\left|{\vec{x}}_{f}-{\vec{x}}_{s}\right|$ is the distance between the source and the pixel in the field map scaled by the wavelength, and $\vec{n}=\frac{{\vec{x}}_{f}-{\vec{x}}_{s}}{\left|{\vec{x}}_{f}-{\vec{x}}_{s}\right|}$ is the normalized vector pointing from the source point to the pixel. All contributions to one pixel originating from different source points (typically 10^5^ contributions per pixel) were averaged into one field vector approximating the electrical field with full phase information at the center of each pixel. The square of the above field vector, $I={\vec{E}}^{2}$, is a relative intensity proportional to the physical intensity; the factor of proportionality arising from all the constants explicitly or implicitly omitted above. To calculate voxel sizes as a function of the laser power, this intensity distribution needs to be correlated to the laser power. We start by expressing the latter as a factor f times the threshold laser power (determined to be 9.3 mW by the analysis of the observed pillar diameters according to Equation (1); see Figure 1b). On the other hand, the above intensity distribution has a maximum $I_{max}$ at the focal spot. At the threshold laser power (f=1), this maximum is equivalent to the polymerization threshold $I_{th}$, $I_{max}=I_{th}$. At a higher laser power, the whole intensity distribution is multiplied by f, and polymerization is initiated wherever the scaled intensity exceeds the threshold. The theoretical height of a voxel is thus determined as the size of the interval in a longitudinal section through the intensity map where $f\cdot I>I_{th}$. To derive the width of the voxel, the same analysis is applied to the cross-section.

The resulting theoretical voxel sizes were used to calculate the theoretical height h of the pillars as follows:

(7) h=(a−1)b+c+d/2

where a=4 is the number of stacked voxels, b = 300 nm is the vertical center-to-center distance between voxels, c=−200 nm is the centre distance of the first voxel from the substrate interface, and d is the height of an individual voxel obtained from the numerical simulations. The resulting theoretical pillar heights and diameters were further used to calculate the initial aspect ratios of pillars and to estimate the shrinkage by comparing the theoretical with the experimental values (see Table S1 in the Supplementary Materials).

### 3.2. Optical Spectra

Although no reflection spectra were recorded due to instrumental limitations, an attempt was made to correlate the observed colors with simulated optical spectra. These were calculated from simulated diffraction efficiencies taking into account the different diffraction orders and the finite aperture angle of the microscope objective used. Diffraction was simulated by rigorous coupled-wave analysis (RCWA) using the electromagnetic solver, S4, developed at the Stanford University [39,40]. In these simulations, the pillar shape was approximated as a cylinder with an ellipsoidal tip. The radii of the cylinders $r_{c}$ were set to the experimentally determined radii of pillars fabricated at different laser powers. The cylinder heights $h_{c}$ were calculated with $h_{c}\;=\;h_{p}-h_{e}$, where $h_{p}$ was the measured pillar height. The height of the ellipsoidal tip $h_{e}$ was assigned to half the voxel height evaluated by Monte Carlo simulations described above, as this parameter was hard to determine experimentally. Using this set of parameters, the pillar envelope function can be described as follows:

(8) $$y\left(z\right)=\{\begin{array}{cc} r_{c} & \mathrm{for}\;z\leq h_{c}\\ r_{c}\sqrt{1-\left(\frac{z-h_{c}}{h_{e}}\right)} & \mathrm{for}\;h_{c}<z\leq h_{p} \end{array}$$

where the z-axis is assumed to be normal to the substrate surface, and thus corresponds to the structure expansion in the vertical direction. The pillars were discretized in slices of 10 nm. A periodic boundary condition was applied with a box size of 1 × 1 µm (in accordance with the pillar center-to-center distance) as well as an incidence angle of 0°. The refractive index and extinction coefficient of the cured photoresist and the fused silica substrate were taken from ellipsometry measurements (see Figure S1 in the Supplementary Materials).

In experiments, the observed colors originate from reflected light that is diffracted by the grating. Reflection occurred at two different interfaces, i.e., the pillar-substrate interface and the backside of the 1 mm fused silica substrate. Due to the opening angle of the objective with NA = 0.45, only diffraction angles of equal to or below 27° were collected. To quantify the contribution of different diffraction orders to the coloration, an overlap factor OF between the incident light and the light cones of different diffraction orders were numerically calculated using the given geometrical parameters of the fabricated nanostructures and the imaging system (angle of incidence, collection angle). In the case of the 1st diffraction order, OF was found to range from 0.45 at a wavelength of 400 nm to 0.045 at 800 nm. Overlap factors for higher diffraction orders were zero except for negligible values at the shortest wavelengths and, therefore, were not considered in further calculations. The reflection at the pillar-substrate interface was calculated by summing 100% of the reflectivity values obtained for the 0th diffraction order and the values of the 1st diffraction order multiplied by $4OF_{\lambda }$, where 4 is the number of contributing 1st order diffraction cones, and $OF_{\lambda }$ the overlap for the respective wavelength. The reflection at the backside of the substrate was calculated in the same way but using transmissive diffraction efficiencies. For each grating, both contributions were added to a final reflection spectrum.

## 4. Results and Discussion

### 4.1. Nanopillar Sizes

Sizes of the nanopillars fabricated with laser powers varying from 10 to 23 mW were obtained from SEM-micrographs (Figure 1a). The pillar diameters ranged from 120 nm to 430 nm (measured at the bottom of the pillars). The heights of the pillars extended from 330 to 1315 nm. The structures exhibited an almost constant aspect ratio (height over diameter) of about 3 (Figure 1b). Evaluation of the defect rate revealed high quality of the obtained optical gratings with 100% upright standing pillars for all gratings with pillar diameters down to 184 nm (Figure 1c). Gratings fabricated with the lowest laser power of 10 mW and thus, closest to the polymerization threshold, exhibited 30% freestanding pillars and 70% collapsed structures. With a diameter of 120 nm, these pillars were the smallest high aspect ratio pillars fabricated with TPL to our best knowledge so far. The collapse of the pillars is most likely induced by capillary forces during drying upon development and post-curing. These collapses occur when the capillary forces exceed the elastic restoring forces of the pillars [36]. As the latter decrease with pillar diameter, smaller structures tend to collapse more easily. For the sake of completeness and to demonstrate the importance of using a UV-post-curing during development, similar pillar structures were fabricated without the additional UV-exposure, exhibiting much more defects even for larger structures (see Figure S2 in the Supplementary Materials).

Theoretical voxel sizes obtained from numerical simulations ranged from 144 to 503 nm in diameter and from 531 to 1873 nm in height with a mean aspect ratio of 3.6 ± 0.1. As a result, the theoretical height of the nanopillars (i.e., four vertically stacked voxels in accordance to Equation (7)) ranges from 965 to 1637 nm as shown in Figure 1b. All values are summarized in Table S1 in the Supplementary Materials. For the diameter, the numerical simulations overestimate the experimental data by 18 ± 3% for all applied laser powers. For the heights, the numerical values overestimate the experimental data by 65% for 10 mW, but only 20% for 23 mW. These discrepancies are most likely related to the shrinkage of the nanopillars, their adhesion to the substrate, and surface tension [36,41]. The degree of shrinkage is mostly related to the removal of unreacted molecules during development. With the reduction of the laser power, the amount of unreacted and not covalently bound oligomers and fragments increased, which in turn did not contribute to the formation of the nanostructures. Furthermore, the distance of the stacked voxels was kept constant for all laser powers, so that voxels overlapped less at lower laser powers, which amplified the effect of an incomplete cross-linking reaction. These unreacted and non-crosslinked fragments were removed during development, which explains higher shrinkage with lower laser power. Upon development, the nanostructures remained in isopropanol and were exposed to UV again. This post-curing led to an additional crosslinking reaction providing enhanced mechanical stability and higher resistance against a capillary forces during drying as is evident from the comparison with structures developed without the additional cross-linking (Figure S2 in the Supplementary Materials). Furthermore, the shrinkage close to the substrate is limited by the adhesion of the nanopillars to the rigid substrate, which led to low shrinkage of the nanopillar diameters. The shrinkage of the pillars heights, in contrast, is not constrained and thus much stronger. This anisotropic shrinkage as well as surface tension effects are assumed to lead to the conical shape of the nanopillars.

### 4.2. Optical Properties

**Figure 2:**
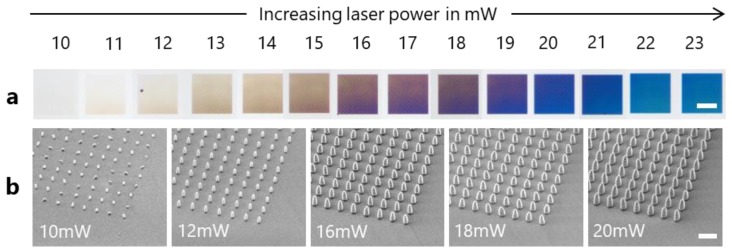
Optical appearance of the nanopillar gratings in dependence on the laser power. (**a**) Optical micrographs of 50 × 50 µm nanopillar gratings on a fused silica substrate. Scale bar is 25 µm. (**b**) Scanning electron micrographs showing the corresponding nanopillars. Scale bar is 1 µm.

**Figure 3:**
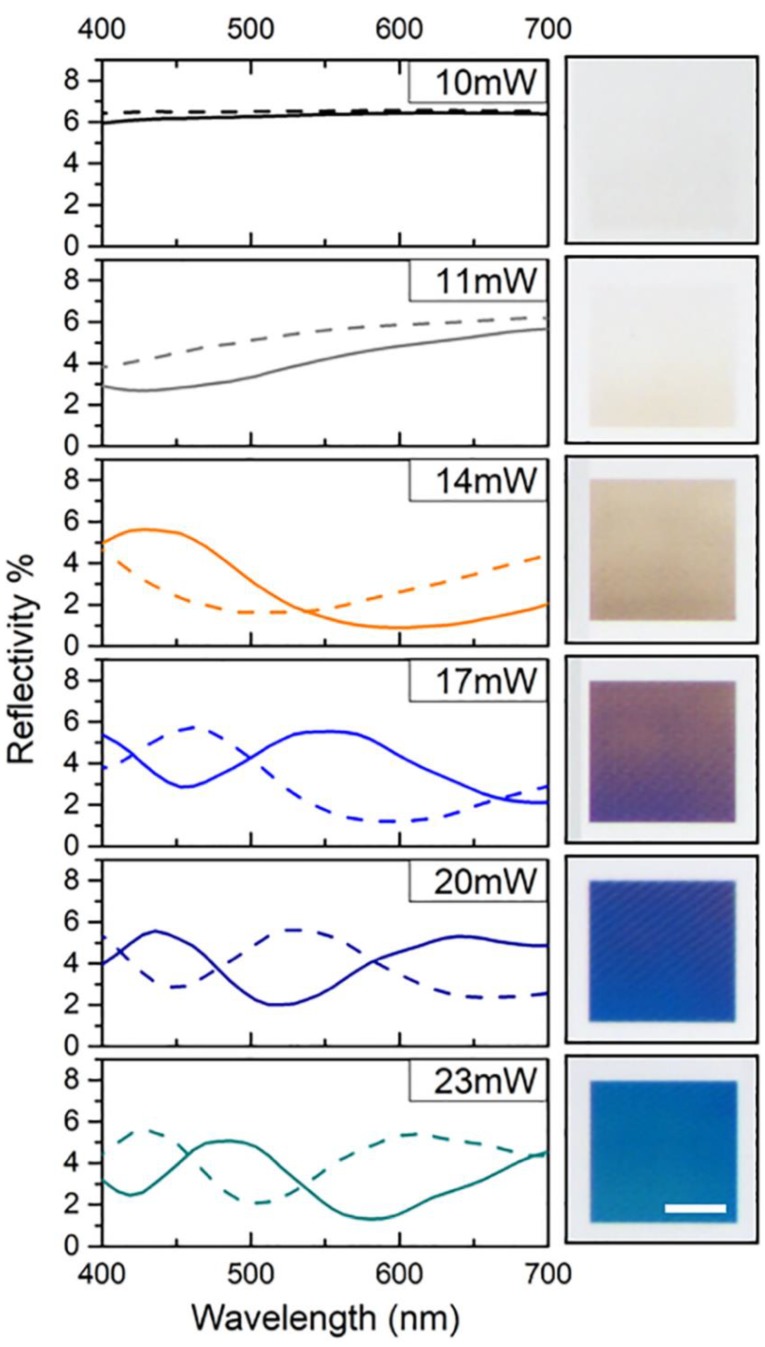
Optical properties of the nanopillar gratings. Reflectivity in dependence on the wavelength obtained from numerical simulations for structure sizes as measured by SEM (solid line) and pillars assuming 20% smaller diameters (dashed line) (left) compared to optical micrographs of nanopillar gratings fabricated with different laser powers (right). The scale bar is 25 µm.

Figure 2 shows optical micrographs of the nanopillar gratings. The divergence angle of the illuminating light as well as the collection angle of the microscope were both 27°. The optical micrographs revealed a colored reflection of the gratings, which is based mainly on diffraction and interference effects. Absorption can be neglected due to the low extinction coefficients of all materials involved (Figure S1 in the Supplementary Materials). The color of the gratings changed with the laser power from slight brownish (11 mW) to blue-green (23 mW). For 10 mW, the surface appeared colorless (Figure 2a). To quantify the optical appearance of the gratings, the optical micrographs were compared with numerical simulations that provide expected optical spectra for the experimental set-up used (Figure 3). The simulations were performed for six gratings corresponding to 10 mW (colorless), 11 mW (slightly yellow), 14 mw (brown), 17 mW (blue-brown), 20 mW (blue), and 23 mW (blue-green).

The obtained spectra did not fully agree with the colors recorded by optical microscopy. Minor deviations can be expected from using diffraction efficiencies for normal incidence to approximate the whole cone of incident light. Moreover, considerable differences could be attributed to variations of the pillar shape. In the fabricated gratings, the shape of the pillars varied between cylinders and cones (compare Figure 1a), whereas cylindrical pillars with elliptical tips were assumed in the simulations. The shape, though, is important for the choice of an appropriate pillar diameter for simulations. This argument was confirmed by simulations with 20% smaller pillar diameters, which led to a significant blue-shift of the spectra (dashed lines in Figure 3) and a better correspondence with the colors observed. From this, it can be concluded that even small variations in the shape of the pillars dramatically affect the optical appearance of the gratings, and that it is therefore not sufficient to evaluate diameters at the pillars’ bases only. This outcome supports our arguments that good understanding of occurring effects, such a shrinkage, surface tension, and related mechanisms, as well as their influence on the feature shape and sizes are important for a precise prediction of the optical properties of pillar gratings fabricated via TPL.

The total reflectivities shown in Figure 3 comprise the reflected diffraction at the pillar-substrate interface as well as the transmissive diffraction of light reflected at the backside of the substrate. As these two are expected to differ strongly in their minima due to variation in the optical path, minima as low as 1% predicted by simulation were surprising. Nevertheless, their presence is confirmed by the intense colors of the gratings. We assume that this could be caused by Mie resonances [42]. This assumption is supported by the strong dependence of the spectra on the pillar diameter. Small pillars obtained at low laser powers interact predominantly with ultraviolet to blue light, leading to a brownish hue. As the pillar size increases with increasing laser power, the resonance shifts towards red wavelengths, resulting in a blueish hue. This variability in color due to the size and shape of nanopillars gratings allows for efficient diffractive color filters.

## 5. Conclusions

Optical pillar gratings were successfully fabricated via TPL at different laser powers close to the polymerization threshold of the photoresist and investigated with respect to their sizes and optical properties using imaging techniques and numerical simulations. The following conclusions can be drawn:

(1) Defect-free nanopillar gratings were fabricated down to pillar diameters of 184 nm and aspect ratios about 3. The smallest pillar diameters achieved were 120 nm, but on imperfect arrays, and therefore would require further optimization in fabrication.

(2) Simulations of the voxel sizes overestimated experimental pillar sizes by 20% in lateral and up to 65% in the vertical direction. This effect can be rationalized by shrinkage that differs due to varying amounts of unreacted oligomers, different overlaps between adjacent voxels, substrate adhesion, and probably surface tension effects.

(3) The nanopillar gratings interfered with visible wavelengths and varied in their optical properties depending on the pillar sizes tuned by TPL. The simulation of the optical spectra confirmed that the coloration originates indeed from the diffraction of reflected light, but also prompts the notion that the size and shape of the nanopillars strongly influence the optical appearance.

In summary, optical gratings based on different pillar sizes can be manufactured by varying the laser power in TPL in a single process step on one surface. However, the fabrication of precise optical gratings close to the polymerization threshold of the photoresist requires a deep understanding of the involved processes.

## Supplementary Materials

The following are available online at https://www.mdpi.com/2079-4991/9/10/1495/s1, Table S1: Summary of pillar dimensions as a function of the laser power. Figure S1: Optical properties of the cured photoresist and the fused silica substrate obtained by ellipsometry. Figure S2: Optical pillar gratings when fabricated without UV-post-curing.
