# Supplementary material for: Nanopillar Diffraction Gratings by Two-Photon Lithography
Source: Nanomaterials (Basel). 2019 Oct 19;9(10):1495. doi: 10.3390/nano9101495 (PMC6836244; doi:10.3390/nano9101495)
Supplement: Supplementary file 1 [file nanomaterials-09-01495-s001.pdf]

Supplementary Information

Nanopillar Diffraction Gratings by Two-Photon Lithography

Julia Purtov [1,2,*], Peter Rogin [1], Andreas Verch [1], Villads Egede Johansen [3], and René Hensel [1,*]

[1] INM—Leibniz Institute for New Materials, Campus D2 2, 66123 Saarbrücken, Germany; Peter.Rogin@leibniz-inm.de (P.R.); Andreas.Verch@leibniz-inm.de (A.V.)

[2] Department of Materials Science and Engineering, Saarland University, 66123 Saarbrücken, Germany

[3] Department of Chemistry, University of Cambridge, Lensfield Road, Cambridge CB2 1EW, UK; villads.egede.johansen@gmail.com (V.E.J.)

* Correspondence: julia-purtov@web.de (J.P.); rene.hensel@leibniz-inm.de (R.H.)

Table S1. Summary of pillar dimensions as a function of the laser power. Experimental data were obtained from scanning electron microscopy (SEM) micrographs.

	SEM-investigation			Fit Sun et al.	Numerical Simulations				
Laser Power [mW]	Pillar Diam. [nm]	Pillar Height [nm]	Fraction of upright pillars [%]	Voxel Diam. [nm]	Voxel Diam. [nm]	Voxel Height [nm]	Pillar Height [nm]	Aspect ratio	Shrinkage [%]
10	120 ± 11	333 ± 13	29.45	115	144	531	965	6.69	65.5
11	184 ± 5	581 ± 30	100	178	230	781	1.09	4.74	46.72
12	225 ± 7	660 ± 14	100	221	275	959	1.18	4.29	44.04
13	230 ± 14	822 ± 39	100	254	312	1.113	1.257	4.02	34.58
14	284 ± 7	868 ± 28	100	281	344	1.228	1.314	3.82	33.94
15	298 ± 6	935 ± 31	100	303	370	1.33	1.365	3.69	31.5
16	313 ± 12	1.054 ± 32	100	323	392	1.424	1.412	3.6	25.35
17	336 ± 13	1.099 ± 17	100	341	413	1.502	1.451	3.51	24.26
18	358 ± 25	1.085 ± 36	100	357	433	1.576	1.488	3.44	27.08
19	376 ± 9	1.21 ± 33	100	372	449	1.664	1.532	3.41	21.02
20	379 ± 26	1.224 ± 44	100	385	464	1.708	1.554	3.35	21.24
21	411 ± 17	1.193 ± 39	100	397	478	1.768	1.584	3.31	24.68
22	410 ± 18	1.236 ± 24	100	408	491	1.825	1.613	3.29	23.35
23	427 ± 20	1.315 ± 35	100	419	503	1.873	1.637	3.25	19.65

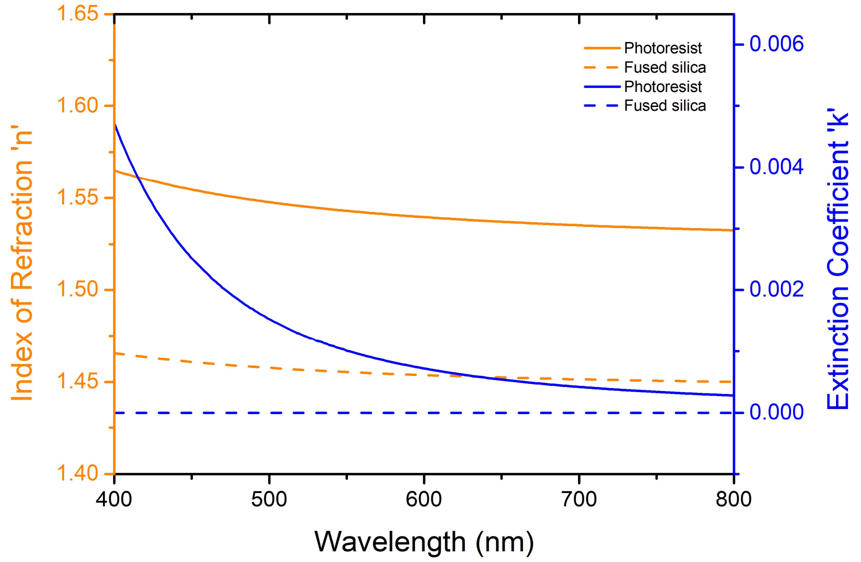

Figure S1. Optical properties of the cured photoresist and the fused silica substrate obtained by ellipsometry.

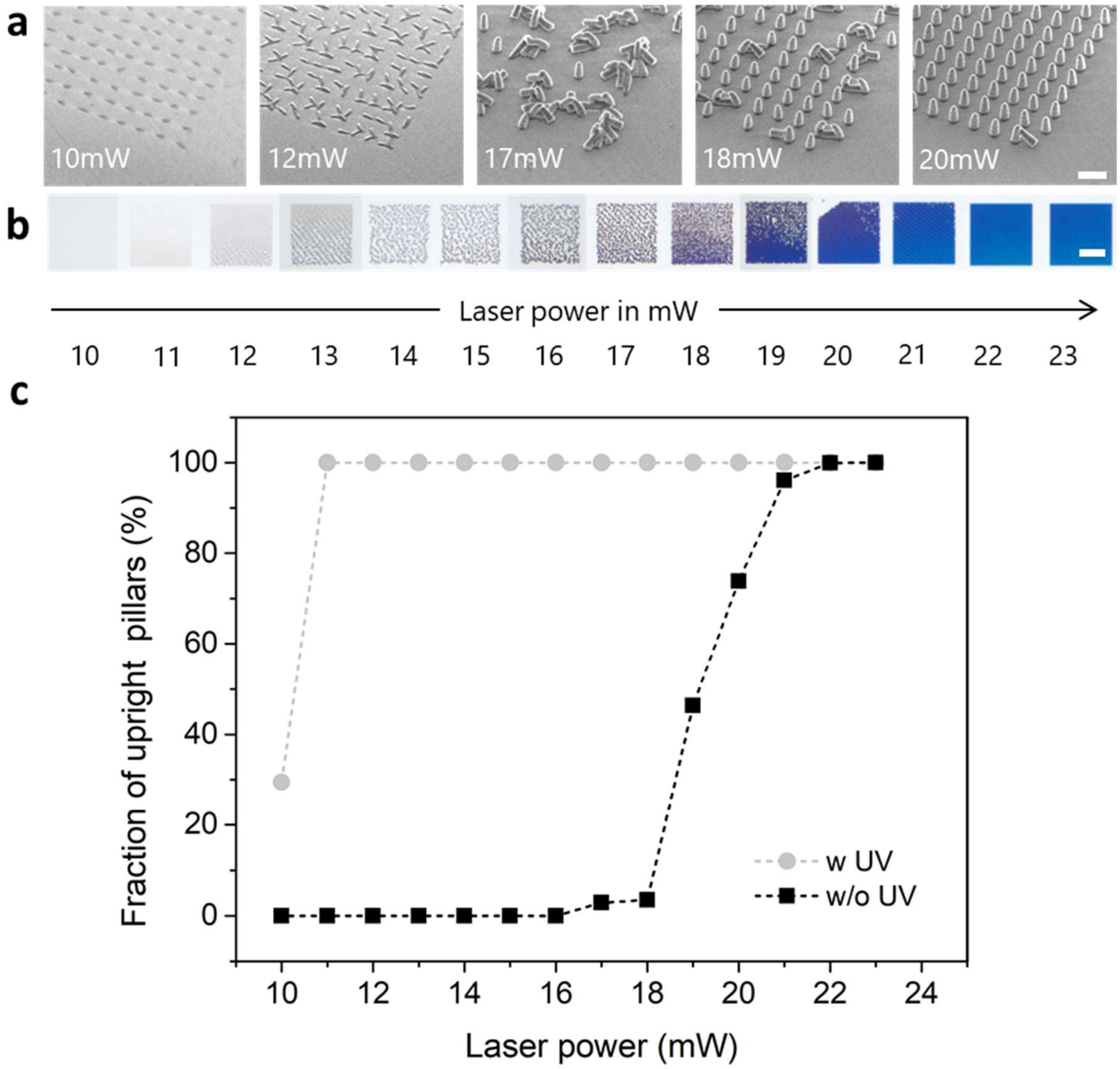

Figure S2. Optical pillar gratings when fabricated without UV-post-curing. (a) SEM-micrographs. Scale bar is 1 µm. **(b)** Optical micrographs of 50x50 µm nanopillar gratings on a fused silica substrate. Scale bar is 25 µm. **(c)** Fraction of upright pillars without (black squares) and with additional cross-linking (grey circles). Dotted lines are added to guide the eyes.
